# Designing phase II trials in cancer: a systematic review and guidance

**DOI:** 10.1038/bjc.2011.235

**Published:** 2011-06-28

**Authors:** S R Brown, W M Gregory, C J Twelves, M Buyse, F Collinson, M Parmar, M T Seymour, J M Brown

**Affiliations:** 1 Clinical Trials Research Unit, University of Leeds, Leeds LS2 9NG, UK; 2 Leeds Institute of Molecular Medicine, University of Leeds and St James's Institute of Oncology, Level 4, Bexley Wing, St James's University Hospital, Beckett Street, Leeds, LS9 7TF, UK; 3 International Drug Development Institute, 30 Avenue Provinciale, 1340 Louvain-la-Neuve, Belgium; 4 Medical Research Council Clinical Trials Unit, Stephenson House, 158-160 North Gower Street, London NW1 2ND, UK

**Keywords:** phase II, methodology, systematic review, trial design, guidance manual

## Abstract

**Background:**

Literature reviews of cancer trials have highlighted the need for better understanding of phase II statistical designs. Understanding the key elements associated with phase II design and knowledge of available statistical designs is necessary to enable appropriate phase II trial design.

**Methods:**

A systematic literature review was performed to identify phase II trial designs applicable to oncology trials. The results of the review were used to create a library of currently available designs, and to develop a structured approach to phase II trial design outlining key points for consideration.

**Results:**

A total of 122 papers describing new or adapted phase II trial designs were obtained. These were categorised into nine levels, reflecting the practicalities of implementation, and form a library of phase II designs. Key design elements were identified by data extraction. Along with detailed description of the key elements and the library of designs, a structured thought process was developed to form a guidance document for choice of phase II oncology trial design.

**Conclusion:**

The guidance offers researchers a structured and systematic approach to identifying phase II trial designs, highlighting key issues to be considered by both clinicians and statisticians and encouraging an interactive approach to more informed trial design.

Over recent years there has been the emergence of a wide range of new cancer therapies, with highly variable modes of action. The pathway of development of novel therapeutic agents is well established (Figure 1). It typically takes up to a decade from preclinical development to new drug approval, and is estimated to cost hundreds of millions of dollars, although the average amount is difficult to accurately estimate (Collier, 2009). Only a small number of newly licensed therapeutic agents are related to oncology each year, and cancer drug attrition rates are significantly higher than in other therapeutic areas, with success in 5% of cancer drug developments compared with 20% in cardiovascular drug development, and improvements are urgently required (DiMasi and Grabowski, 2007; Walker and Newell, 2009).

These depressing statistics have led to increased attention on the drug development process, aiming to identify ways of reducing the attrition rate. Phase II trials act as screening tools to assess whether a treatment has sufficient activity to warrant further investigation in large costly phase III trials. In this respect, the term ‘activity’ is used to describe the ability of an investigational treatment to produce an impact on a short-term or intermediate clinical outcome measure; in oncology, this is commonly either response rate or proportion of patients progression-free at a specific time point, but in the era of targeted therapies this may be measured as a change in biomarker level. With increasing costs of clinical trial design and implementation, including the significant costs associated with phase II and phase III studies, it is essential that resources are appropriately streamlined. Phase II studies must be designed, performed and reported to allow accurate interpretation of results and to obtain the best quality data to facilitate and inform unbiased decisions regarding the subsequent development of the drug(s) under study in the phase III setting.

It is important for researchers designing phase II trials (clinicians and statisticians alike) to understand the elements of phase II design. Given the number and variety of designs available, it is also essential that the most appropriate design is selected to accommodate the specific requirements of a trial, and to allow the most informative data to be obtained. Several reviews have discussed the current use of statistical designs in published phase II studies in cancer (Mariani and Marubini, 2000; Perrone *et al*, 2003; Thezenas *et al*, 2004; Lee and Feng, 2005). The review by Mariani and Marubini (2000) was restricted to articles cited on MEDLINE that were published in 1997, aiming to identify which statistical methodologies were in current use in cancer-specific phase II studies. Of 295 single-arm trials using objective response rate, only 58 (20%) reported an identifiable statistical design. The most common statistical approach was the two-staged hypothesis testing method, with Simon's methods (optimal and minimax) being the preferred methods. Gehan's two-stage design was the most frequently referenced design. The low frequency of an identifiable statistical design was acknowledged as a remarkable weakness of the studies reviewed, as well as the poor quality of reporting of statistical design where it was identifiable, and the low level of compliance with the design in the study conduct and result interpretation. It was concluded that there was a need for better understanding of available statistical designs for phase II studies in cancer, to increase the quality of studies, and also the need for better reporting.

These results have been borne out in other reviews. Lee and Feng (2005) published a review of statistical designs utilised between 1986 and 2002 (*n*=266), and reported that statistical design was only documented in 46% of cases, with only 23% providing sufficient data to characterise the design used. A similar exercise reported in breast cancer phase II studies published between 1995 an 1999 in seven oncology journals (impact factor ⩾2), again found limited reporting of statistical design (51out of 145, 35.5% Perrone *et al*, 2003).

Although guidance exists for specific elements of phase II design, such as when to incorporate randomisation, choice of outcome measures and broad design categories (Mariani and Marubini, 1996; Booth *et al*, 2008; Seymour *et al*, 2010), there is little information to assist trialists and clinicians in choosing between specific statistical designs or adaptations/extensions to commonly used designs. None of the available literature provides a contemporary practical guide to phase II design.

A systematic literature review was performed of phase II clinical trial methodology to produce a detailed library of available phase II designs applicable to cancer trials. This provided the background information to facilitate the development of a phase II trial guidance manual aiming to generate a structured and systematic process to categorise and incorporate the principles behind the different designs, assisting researchers in their choice of phase II design. We describe the methodology and results of the systematic literature review and categorisation, and briefly introduce the content and layout of the subsequent phase II trial design guidance manual. The manual is freely available to researchers upon request via the University of Leeds Clinical Trials Research Unit (CTRU) website (http://ctru.leeds.ac.uk/phaseII).

## Materials and methods

A systematic literature review was conducted to identify literature detailing phase II trial designs currently available.

### Literature search

**Figure 2:**
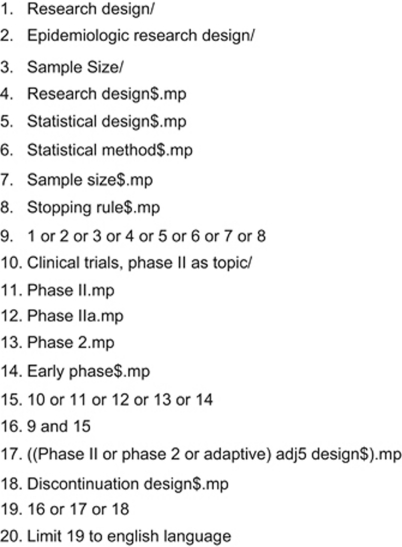
Search terms to be used in MEDLINE for the systematic literature review of statistical methods for the design of phase II studies in cancer. Key: .mp=title, original title, abstract, name of substance word, subject heading word; $, wildcard; adj*n*, within *n* words either side; /, MeSH term all subheadings.

Data were identified by searches of MEDLINE, EMBASE, Current Index to Statistics and Science Citation Index indexed by Web of Science. The search strategy implemented for the MEDLINE database is detailed in Figure 2. Search strategies were developed for each database because of the differing format and searching processes for each database. As the intention of the systematic literature review was to be thorough but not exhaustive, a further review of abstracts and reports from meetings, and hand searching of key journals, was not incorporated. The databases were searched for material published on each database till 9/10 February 2008. Additional papers were subsequently identified via MEDLINE auto-alert up to January 2010 and incorporated into the library of designs.

### Eligibility criteria

Each record identified via the search strategy was assessed for suitability. Full articles of all potentially useful publications were obtained. Articles that could not be identified on-line or through the University of Leeds library were classed as unobtainable. A random selection of 20 citations that were not selected for full-text review was independently reviewed by two other members of the research team to confirm their exclusion.

Papers specifically discussing the advantages and disadvantages of previously published designs, that is, not outlining new or adapted designs, were separated as discussion papers. Papers were included which referred to statistical methodology for phase IIa and IIb studies, and phase II/III trials and/or which discussed the issues surrounding randomisation in phase II studies. Papers were excluded which referred to statistical methodology for the design and analysis of feasibility or pilot studies, or phase I, III and IV studies, if the methodology described was applicable only to disease areas other than cancer, if the statistical methodology was described for the analysis of phase II trials only (as opposed to design) or if the paper discussed a randomisation method only. Any papers that were not relevant to the review, for example, phase II trial results only, were also excluded, as were conference abstracts with no further information available.

### Paper selection

Retrieved full-text articles were reviewed and the inclusion/exclusion criteria applied by one of the authors (SB). From each paper included, data were extracted using a specifically designed data extraction form. During the development of the data extraction form, nine broad categories of phase II trial designs were identified: one-stage, two-stage, multi-stage/group sequential, decision theoretic, continuous monitoring, three-outcome, phase II/III, response adaptive randomisation and randomised discontinuation. These design categories were used to group the papers, with some papers being represented in more than one category. Papers describing adaptive designs were incorporated in the review where they specifically related to phase II design. ‘Adaptive design’ has not been incorporated as a specific design category here as it was felt that this formed a subset of the categories already established, for example, a two-stage design may incorporate adaptations at the end of stage one. Previous review papers have used alternative grouping categories for phase II designs, either focussing on single-arm *vs* randomised studies (Seymour *et al*, 2010), or for those of molecularly targeted agents only, considering design categories such as randomised designs, enrichment designs and adaptive Bayesian designs (Booth *et al*, 2008). In contrast to these reviews, the chosen categories focus more on the practical identification and implementation of trial designs, reflecting trial design rather than trial analysis.

Other information was collected regarding the aim of the phase II trial; end points considered; parameters required for the design; sample size; number of treatment arms; randomisation; whether a design was adaptive; analysis; and computational and software requirements. Given the nature of the review and the information being sought, a data extraction form alone was deemed insufficient to inform the guidance document, therefore free text summaries of papers included after the full-text review were also incorporated. An assessment of ease of implementation of each design paper was made. Designs were defined as not being easy to implement if any of the following held: data required for implementation not likely to be available; sample size justification not provided; no decision criteria detailed; assessment of every patient is required before the next patient can be recruited; software is required to implement/analyse the design, which is not noted to be available and insufficient detail is given to allow implementation. These criteria reflect the ability to use the design paper mostly in its raw format to design and implement a phase II oncology trial.

Data extracted was recorded in an excel spreadsheet and read in to SAS version 9.2 (SAS Institute Inc., Cary, NC, USA) for data summary. Easily implementable design papers were summarised descriptively and grouped as per their primary design category, with the detailed narratives for each paper forming the basis of the phase II trial design library.

## Results

**Figure 3:**
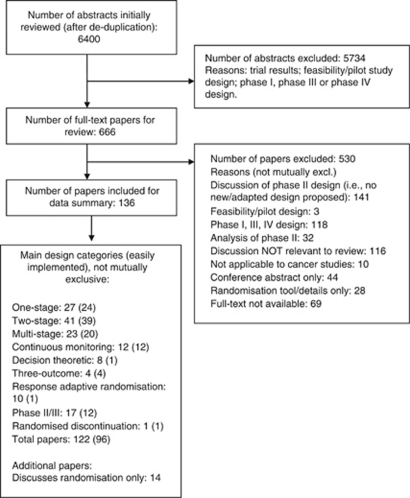
Flow diagram of papers in review.

After removal of duplicate papers, the literature search identified 6400 abstracts, from which 666 full-text papers were retrieved. Review of full-text papers identified 136 papers as eligible. Figure 3 displays the number of citations, full-text papers and designs identified from the review through each stage. The majority of papers excluded at the first stage were due to papers describing only the results of a phase II trial, rather than the specific design methodology.

Of a total of 136 papers included for data extraction, 14 papers discussed randomisation issues only. The remaining 122 papers can be broken down as follows (not mutually exclusive): one-stage 27 papers; two-stage 41 papers; multi-stage/group sequential 23 papers; continuous monitoring 12 papers; decision theoretic 8 papers; three-outcome 4 papers; response adaptive randomisation 10 papers; phase II/III 17 papers; and randomised discontinuation 1 paper. In the flow diagram, the numbers in brackets represent the number of papers within each design category that have been classed as easy to implement. Of the 122 papers discussing any of the nine top-level design categories, 96 papers were classed as being easy to implement. The designs described by these 96 papers, and the corresponding narrative summaries, formed the library of phase II trial designs around which the guidance manual was developed. The library was subsequently updated with designs identified via MEDLINE auto-alerts to incorporate papers listed to January 2010.

### Developing the manual

**Figure 4:**
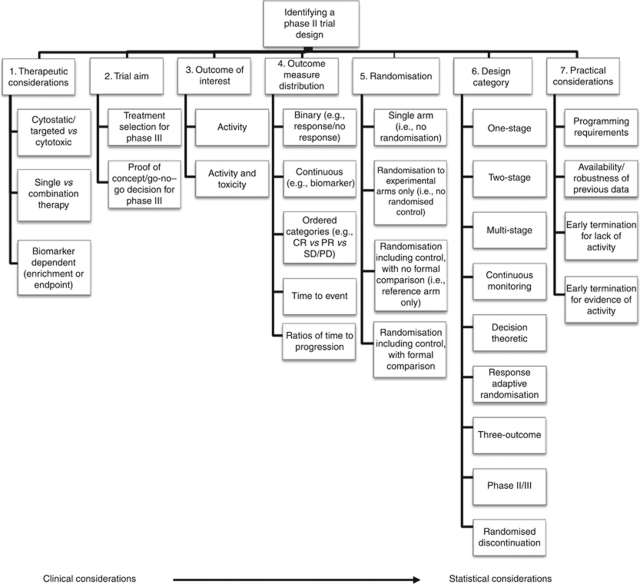
Flow diagram of phase II trial design thought process.

Issues that underlie the thought processes influencing researchers when choosing between clinical trial designs were distiled from the systematic literature review, but also from previous reviews and discussion papers on specific elements of phase II trial design (Booth *et al*, 2008; Adjei *et al*, 2009; Dhani *et al*, 2009; Rubinstein *et al*, 2009; Seymour *et al*, 2010). These key elements of phase II design were grouped into seven categories, as displayed in Figure 4.

#### Therapeutic considerations

Therapeutic considerations represent broad clinical issues, which should be discussed with the clinical investigators at the first phase II trial meeting between the clinician and statistician, the outcome of which will inform decisions to be made later in the design process. The mechanism of action of the treatment is perhaps the most important. This will inform the types of outcome measures that may be used in the trial, and whether or not randomisation should be incorporated. For cytotoxic agents tumour shrinkage is widely accepted as reflecting anticancer activity. Many new cancer therapies are, however, targeted at specific molecular pathways relevant to tumour growth, apoptosis or angiogenesis and may be cytostatic. In that case, where a cytotoxic effect may not be anticipated, it could be argued that a change in tumour volume may not be an appropriate phase II outcome measure to capture the activity of the treatment(s). Whether the intervention to be studied is a single agent or to be given in combination with another, already established therapy, is important in deciding whether or not to incorporate randomisation. And finally, the use and availability of biomarkers associated with either the experimental treatment or the disease in question will help to inform decisions regarding outcome measures, randomisation and design categories.

#### Trial aim

The NCI Clinical Trial Design Task Force primarily categorises phase II trials as being single arm or randomised. However, as our manual aims to offer a practical approach to the design of phase II trials, the initial grouping category for the trial designs has been taken to be the aim of the trial. Whether the aim is to select the most promising of two or more candidates to take forward or to make a go/no-go decision for a single investigational agent (or combination of agents) allows distinction between selection designs and go/no designs.

#### Outcome of interest

The outcome of interest of a trial will depend on the current evidence base for the experimental and current standard treatments, and/or the stage of development of the experimental treatment, its mechanism of action and potential toxicity. Where the toxicity of an investigational treatment is well known, or thought to be modest in the context of the phase II decision-making process, the primary phase II trial outcome measure will usually be antitumour activity, with toxicity among the secondary outcome measures. If, however, the toxicity profile of an investigational treatment is of particular concern, the activity and toxicity of the treatment may be considered as joint primary outcome measures

#### Outcome measure distribution

Emerging cancer treatments have many differing modes of action and this is reflected in the choice of outcome measures. Specifically, although response probably remains the most widely used outcome measure, non-binary definitions or volumetric measures of response, measures of time to event or continuous markers such as biomarkers may be more relevant when evaluating the activity of targeted or cytostatic agents (Karrison *et al*, 2007; Booth *et al*, 2008; Adjei *et al*, 2009; Dhani *et al*, 2009; McShane *et al*, 2009).

#### Randomisation

Whether a phase II trial should be randomised is frequently a key question and its use is widely debated (Buyse, 2000; Yothers *et al*, 2006; Redman and Crowley, 2007). Randomisation is increasingly incorporated in phase II trials, but its use should be considered critically as it is not always appropriate, and can take many forms (Gan *et al*, 2010; Stewart, 2010). The guidance manual discusses the use of randomisation in detail, providing guidance on the use of single-arm designs, designs with randomisation to more than one experimental treatment and randomisation incorporating a control arm either with or without a formal comparison with the experimental arm.

#### Design category

The designs identified by the systematic literature review were recognised as having similarities as well as differences, and were placed into the nine design categories described earlier. Not all these categories are mutually exclusive. For example, a one-stage trial may incorporate a three-outcome design, or be based on a decision theoretic approach. A description of each of the nine categories is provided in the manual, focusing on the practical implementation of each design. Differences associated with the analysis frameworks of the designs are not discussed and is out with the current remit of the manual.

#### Practical considerations

Finally, in developing a structured approach to selecting a phase II trial design, it became apparent that practical considerations such as the number of patients available and the need for specialist computer programming would aid selection between alternative designs. These elements are discussed for each design listed in the design library.

The manual is freely available to researchers upon request from the CTRU's website (http://ctru.leeds.ac.uk/phaseII), and is compiled as follows. Section 1 lays out the contents of the manual, and section 2 provides an introduction to the background of its development and introduces users to its structure, focus and terminology used throughout. The thought process shown in Figure 4 is also introduced in this section. Section 3 provides detailed discussion of each of the key elements presented in the flow diagram, containing further detail on the background and reasoning behind each of the key elements, and information on the various design options available to researchers. Section 4 presents the library of phase II designs, which are grouped according to the various design elements selected from the flow diagram (e.g., Trial Aim: Treatment selection; Outcome of interest: activity; Outcome measure: Binary; Randomisation: including a control arm; Design category: Two stage). Each design is summarised briefly, with detail regarding the practical considerations of the design and any adaptations that may be incorporated. Finally, Sections 5 and 6 provide references and an index, respectively, to each of the phase II designs listed in Section 4.

As discussed, each of the elements presented in the flow diagram in Figure 4 are described in detail in the guidance manual, and are designed to facilitate interaction between the clinician and statistician throughout the design process. Incorporating the initial clinical information determined through point 1 (therapeutic considerations), researchers may make choices at each stage of the flow diagram (points 2 to 6) regarding the key elements of phase II design. This in turn narrows down the number of designs appropriate to the specific requirements of the trial, and users may incorporate practical issues into their final design choice. The guidance itself is not intended to identify a single trial design that researchers must use; rather, it acts as a resource for identifying designs appropriate to the requirements of each trial from which the researchers can make their choice. This allows the library of designs to be used more in the format of a dictionary as compared with being read from cover to cover.

## Discussion

The systematic literature review has identified a surprisingly large number of phase II trial designs available to the cancer research community; a total of 96 papers describing designs classed as easily implemented. This highlights the need to understand key elements associated with the many different designs available to researchers in order that informed decisions can be made at the design stage.

As discussed in the introduction, a number of review papers exist regarding the use of phase II trial designs, and all highlight the poor reporting of clear, identifiable statistical designs in phase II clinical trials. This frequently leads to uncertainty as to the robustness of results reported. With the poor success rates reported in phase III clinical trials, it is essential that the data used to assess the appropriateness of proceeding with investigation of (an) agent(s) in the phase III setting are accurately interpreted. Although we have not addressed reporting requirements in our research, it is clear from previous reviews discussed that this is only possible with transparent reporting of the statistical methodology used.

Although there are broad categories of trial designs that statisticians may generally be familiar with, there are many adaptations within each of these which may be less familiar, but which may actually be particularly useful given there is no ‘one size fits all’ phase II trial design. The findings of the systematic literature review, in conjunction with findings of previous reviews considering the use of phase II trial designs in cancer, re-enforce the need for researchers to be more informed regarding their design choices, considering all aspects of trial design from trial aims and outcomes, to randomisation and the type of design to use.

The systematic literature review (and in particular the summaries of the designs identified as ‘easily implemented’) was performed to inform the development of a manual to guide the reader through the specific points for consideration when designing a phase II clinical trial. This also provides a framework for categorisation of designs to aid transparent reporting of the statistical methodology used in phase II trials.

The manual does not consider trial analysis. Details regarding the statistical theory of some of the designs have been previously discussed in Mariani and Marubini (1996), Machin and Campbell (2005) and Machin *et al* (2009). Examples of using the manual in practice are provided elsewhere (Brown *et al*, 2011).

This manual is not a prescriptive or didactic document, but instead aims to facilitate and encourage an interactive approach to trial design between clinical researcher and statistician, resulting in better-informed choices. The design methodologies identified in this systematic literature review, in conjunction with data included in other review papers on this topic and discussion papers on specific elements of phase II trial design (Booth *et al*, 2008; Adjei *et al*, 2009; Dhani *et al*, 2009; Rubinstein *et al*, 2009; Seymour *et al*, 2010), were used in part to distil issues that underlie the thought processes influencing researchers when choosing different clinical trial designs. The underlying systematic literature review is sufficiently comprehensive in nature and scope such that, rather than being selected from those designs known already by individual researchers, or those available on standard statistical packages, researchers instead may choose from the full range of currently developed methods. Although it is acknowledged that the assessment of ease of implementation of designs is inherently subjective, the criteria on which this judgment has been made are described and reflect the practicalities of design implementation. It is also recognised that already this review is dated. The manual is designed to be fluid in nature and may be updated as phase II trial designs continue to evolve. Many older trial designs do not detail statistical software or programming applications as being readily available, and where not detailed in the manual, we specifically encourage authors to make such software publicly available. We welcome the input of those using the manual and a feedback resource is available on the CTRU website (http://ctru.leeds.ac.uk/phaseII/feedback). Additionally, we will be administering a follow-up questionnaire to users of the manual to enable us to address whether the manual has resulted in better trial design compared to having not applied this structured approach. This will be administered on a regular basis with timing based on the number of guidance manual requests received.

## Figures and Tables

**Figure 1:**
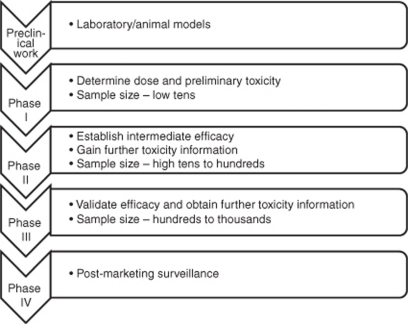
Phases of drug development.
